# Comparative and functional analysis of the digital mucus glands and secretions of tree frogs

**DOI:** 10.1186/s12983-019-0315-z

**Published:** 2019-06-13

**Authors:** Julian K.A. Langowski, Saranshu Singla, Alex Nyarko, Henk Schipper, Frank T. van den Berg, Sukhmanjot Kaur, Henry C. Astley, Sander W.S. Gussekloo, Ali Dhinojwala, Johan L. van Leeuwen

**Affiliations:** 1 Experimental Zoology Group, Department of Animal Sciences, Wageningen University & Research, De Elst 1, Wageningen, 6708 WD The Netherlands; 2 Department of Polymer Science, The University of Akron, 170 University Ave, Akron, Ohio 44325-3909 USA; 3 Biomimicry Research & Innovation Center, Departments of Biology and Polymer Science, The University of Akron, 235 Carroll St., Akron, Ohio 44325-3908 USA

**Keywords:** *Hyla cinerea*, Macrogland, Mucosubstance, Lubrication, Wet adhesion, Cryo-histochemistry, Infrared spectroscopy, Sum frequency generation spectroscopy, Synchrotron micro-computer-tomography

## Abstract

**Background:**

Mucus and mucus glands are important features of the amphibian cutis. In tree frogs, the mucus glands and their secretions are crucial components of the adhesive digital pads of these animals. Despite a variety of hypothesised functions of these components in tree frog attachment, the functional morphology of the digital mucus glands and the chemistry of the digital mucus are barely known. Here, we use an interdisciplinary comparative approach to analyse these components, and discuss their roles in tree frog attachment.

**Results:**

Using synchrotron micro-computer-tomography, we discovered in the arboreal frog *Hyla cinerea* that the ventral digital mucus glands differ in their morphology from regular anuran mucus glands and form a subdermal gland cluster. We show the presence of this gland cluster also in several other—not exclusively arboreal—anuran families. Using cryo-histochemistry as well as infrared and sum frequency generation spectroscopy on the mucus of two arboreal (*H. cinerea* and *Osteopilus septentrionalis*) and of two terrestrial, non-climbing frog species (*Pyxicephalus adspersus* and *Ceratophrys cranwelli*), we find neutral and acidic polysaccharides, and indications for proteinaceous and lipid-like mucus components. The mucus chemistry varies only little between dorsal and ventral digital mucus in *H. cinerea*, ventral digital and abdominal mucus in *H. cinerea* and *O. septentrionalis*, and between the ventral abdominal mucus of all four studied species.

**Conclusions:**

The presence of a digital mucus gland cluster in various anuran families, as well as the absence of differences in the mucus chemistry between arboreal and non-arboreal frog species indicate an adaptation towards generic functional requirements as well as to attachment-related requirements. Overall, this study contributes to the understanding of the role of glands and their secretions in tree frog attachment and in bioadhesion in general, as well as the evolution of anurans.

**Electronic supplementary material:**

The online version of this article (10.1186/s12983-019-0315-z) contains supplementary material, which is available to authorized users.

## Background

Climbing is an important aspect of terrestrial locomotion, as it allows animals to avoid ground-dwelling predators and to access elevated habitats, but poses significant challenges [1], especially on wet and slippery surfaces. For successful climbing, species from various clades have developed a wide range of attachment structures [2]. Studying the various bioadhesive solutions found in nature helps not only to unravel the evolution of the according species [3, 4], but also contributes to the understanding of the fundamental physics and chemistry of attachment, hence providing inspiration for the design of technical adhesives [5].

Traditionally, biological adhesives have been categorised into ‘dry’ (e.g. the ‘hairy’ digital pads of geckos covered with numerous setae; [6, 7]) and ‘wet’ systems (e.g. the adhesive pads of various arachnids, insects, and amphibians such as tree frogs; [8, 9]). Wet adhesives are characterised by the presence of a liquid layer in between the adhesive organ and the substrate, which arguably increases the complexity of the attachment system compared to dry adhesives [8, 10, 11].

Tree frogs, with a snout-vent-length (*ℓ*_SV_) of up to 13.5 cm and a body mass (*m*) up to 160 g, are among the largest terrestrial organisms having wet attachment organs [12, 13], and therefore an interesting group to study the attachment mechanisms in. Generally, amphibians have a moist skin [14] that contains mucus, granular (also serous, poisonous, or venomous), mixed (also seromucous), and lipid glands, which secrete various muco- and other substances [14–17]; see Additional file 1 for the associated mucus nomenclature. The epidermal mucus is involved in various functions including thermoregulation, cutaneous gas exchange, reproduction, and defense against predators and pathogens [14, 16, 18, 19].

The mucus on the adhesive digital pads has been suggested to (i) enable wet adhesion (i.e. capillary and hydrodynamic adhesion; [20, 21]), (ii) lubricate the skin [22] and hence to avoid abrasive wear [16, 23], and (iii) to avoid stiffening, the loss of conformability, and the resulting decrease in attachment performance on rough substrates.

Considering this wide range of potential functions of the mucus in tree frog attachment, surprisingly little is known on the chemical nature of the secreted mucus and on the morphology of the glands that produce it. The glands appear to be clustered in the basal-proximal dermis [24, 25]. Since these early descriptions, the clustered ventral digital glands received to our knowledge little attention. Individual glands are tubuloalveolar with a single-layered cuboidal epithelium [26, 27], covered by a layer of smooth muscle cells [28–30]. Although the glands have been described as mucus glands [27, 29, 31–33], we are not aware of a detailed histochemical characterisation of their secretory content. Also, the exact gland morphology, the gland volumina (i.e. the available amount of mucus), and the distribution of the pores over the ventral epidermal surface (i.e. the spatial distribution of mucus on the pad surface) are unknown.

Here, we present a quantitative analysis of the mucus glands and their secretory products in the digital pads of tree frogs. We focus on *Hyla cinerea* (American green tree frog), one of the most frequently studied tree frog species [21, 27, 29, 34, 35]. By combining synchrotron micro-computer-tomography (µ-CT) and cryo-histochemistry, we study the functional morphology of the dorsal and ventral glands in the digital pads in 3D. Furthermore, we analyse the digital mucus prior to secretion within the glands by cryo-histochemistry as well as after secretion by attenuated total reflectance-infrared spectroscopy (ATR-IR) and interface-sensitive sum frequency generation spectroscopy (SFG), aiming for a characterisation of the chemical composition of the mucus and of its interaction properties at the pad-substrate interface. With these techniques, we compare (i) the mucus from the ventral and the dorsal pad surface in *H. cinerea*, (ii) from the ventral pad surface and the ventral abdomen in the arboreal species *H. cinerea* and *Osteopilus septentrionalis* (Cuban tree frog), and (iii) the abdominal mucus of arboreal (*H. cinerea*, *O. septentrionalis*) and non-arboreal (*Ceratophrys cranwelli*, Pacman frog; *Pyxicephalus adspersus*; African bullfrog) frog species (Additional file 1: Table SI.2). Based on these analyses, we address the following questions:

- Do the ventral mucus glands and their secretions differ from the dorsal mucus and mucus glands in the digital pads? Does the ventral digital mucus differ from the ventral abdominal mucus? And does the abdominal mucus differ between arboreal and non-arboreal frog species? Positive findings on these questions would support a functional specialisation of mucus and glands in tree frogs towards attachment (e.g. by enhancing capillary force generation).
- Can the ventral mucus glands produce enough mucus to fill the pad-substrate gap, which is imperative for wet adhesion, or do tree frogs rely on additional liquid sources?
- How are the ventral digital mucus gland pores distributed across the pad surface?

## Results

### Mucus gland morphology

**Fig. 1:**
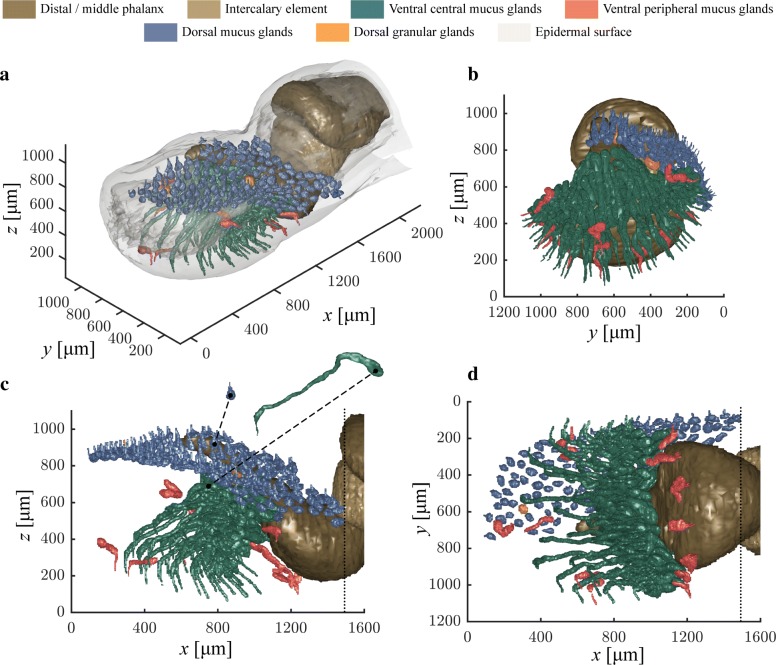
Mucus glands in a digital pad of *Hyla cinerea*. **a** 3D view of the whole digital tip. Only the left half of the approximately bi-laterally symmetric dorsal mucus glands is shown. **b** Frontal, **c** lateral, and **d** ventral view of the glands. The dotted lines indicate the proximal border of gland segmentation. *x*, *y*, *z*: longitudinal, lateral, and vertical spatial coordinates

The digital pads of *H. cinerea* contain several types of glands in different regions of the pad (Fig. 1). Dorsally, the dermal stratum spongiosum contains numerous (*n*=158 for the left half of the dorsal digital cutis) ‘regular’ mucus glands that are evenly distributed across the whole dorsal dermis of the terminal digital segment, with a nearest-neighbour-distance (NND) of 57.3 ±10.2 µm (throughout the manuscript we report mean ± standard deviation unless mentioned otherwise) and a density (*ρ*_d_) of 191.3 gland openings per mm^2^. The dorsal glands have an approximately spherical body with a diameter (*d*_d_) of 45.0 ±5.5µm (assuming spherical gland volumes; Fig. 1) and a volume (*V*_dg_) of 0.050 ±0.019 nL (Additional file 1: Figure SI.1), summing up to a total dorsal gland volume (*V*_d_) of ca. 15.8 nL per pad. The dorsal gland volume per pad surface area (*ρ*_dV_) is 9.5 nL mm^-2^. The gland bodies connect to the dorsal pad surface via short straight ducts, which run approximately perpendicularly to the epidermal surface. Only a few granular glands are present in the dorsal dermis (identified according to [15]).

Ventrally, almost no glands are found in the stratum spongiosum. Only at the proximal, distal, and lateral edges of the adhesive pad, a few glands (*n*=12) are present in the dermal stratum compactum (Fig. 1). The majority of mucus glands (*n*=49) opening to the ventral pad surface is located in the subdermal space delimited by the ventral (and ventrolateral) surface of the terminal phalanx, dorsal and ventral cutaneous collagen, and a collagenous dorsoventral septum running from the distal tip of the terminal phalanx to the ventral cutis (for a detailed description of these structures see [23]). We refer to the combined gland bodies inside this space as ‘gland cluster’.

These ventral mucus glands are clearly tubular, with a mean length (*ℓ*) of 769.2 ±251.2 µm. Their alveolar bodies are elongated—with the longitudinal gland axis running from proximal-ventral towards distal-dorsal—and strongly convoluted. Each gland connects via a thin (*d*≈30 µm) duct to the ventral pad surface. Beginning from the gland body, most ducts turn towards the ventral epidermis just proximally of the dorsoventral septum (Fig. 1b). After piercing the septum, the ducts traverse the internal pad space at an angle of about 45^∘^ with respect to the horizontal plane towards the ventral surface. Upon entering the ventral pad epidermis, the ducts take another sharp turn, such that they run approximately normally towards the pad surface. A single gland has a volume (*V*_vg_) of 0.78 ±0.28 nL, which is significantly higher than that of the dorsal ones (Two-sample t-test, *t*=18.24,*p*<0.001; Additional file 1: Figure SI.1). The total ventral gland volume (*V*_v_) sums up to 38.0 nL. The ventral gland pores have a NND of 120.8 ±27.1 µm, and a gland density (*ρ*_v_) of 52.7 pores per mm^2^ pad surface area. This corresponds with a gland volume density (*ρ*_vV_) of 40.8 nL mm^-2^.

**Fig. 2:**
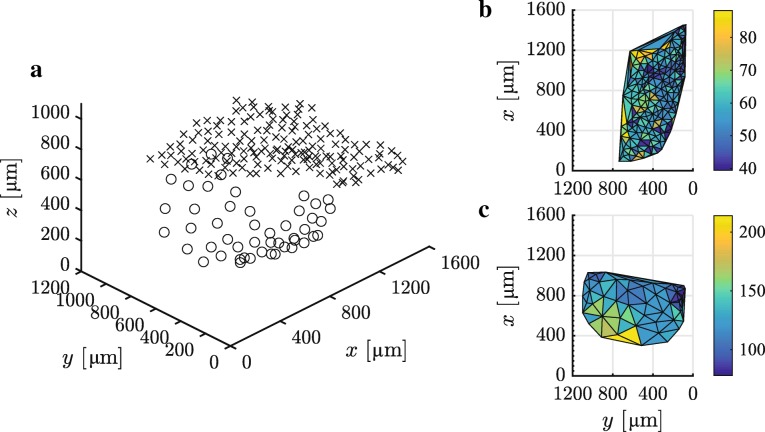
Spatial distribution of the digital mucus gland pores in *Hyla cinerea*. **a** 3D view of the dorsal (crosses; only the left half of the dorsal mucus pores is shown) and ventral (circles) pores on the digital pad cutis. **b** Spatial variation of the dorsal and **c** ventral mucus pore density, approximated by the nearest-neighbour-distance NND [colour-coded in µm], across the pad surface in dorsal view

The dorsal pore NND, an inverse proxy of the pore density, decreases along the lateral pad axis towards the sagittal pad plane (*t*[155] =5.16,*p*<0.001), whereas such a trend was not observed for the longitudinal pad axis (*t*[155] =1.82,*p*=0.07, Fig. 2). For the ventral glands, NND significantly increases along the lateral (*t*[46] =6.30,*p*<0.001) and decreases along the longitudinal (*t*[46] =−9.55,*p*<0.001) pad axis. The change in NND, and hence in pore density, is stronger along the longitudinal (0.11 µm µm^-1^) than along the lateral pad axis (0.04 µm µm^-1^), with an increasing pore density towards the proximal end of the digital pad.

Immunohistochemical staining shows that both dorsal and ventral glands are ensheathed by myoepithelial cells (Additional file 1: Figure SI.3), visible as brown fibres containing elongated, relatively thick nuclei. The myoepithelial cells of the dorsal glands appear thicker and are more intensively stained than those of the ventral glands.

### Mucus gland cryo-histochemistry

**Fig. 3:**
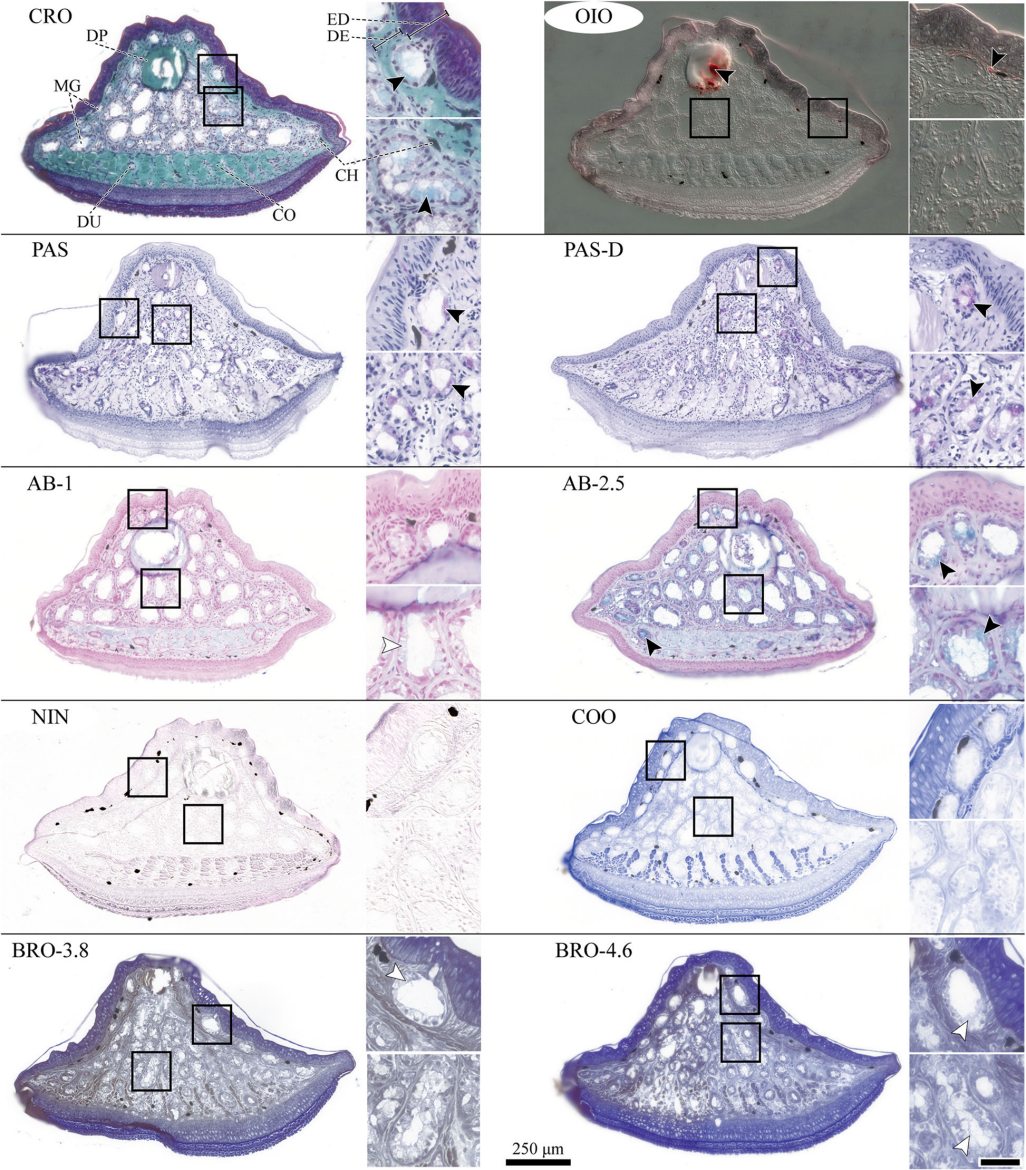
Brightfield micrographs of cryo-histochemically stained mucus glands in the digital pads of *Hyla cinerea*. Per staining, an overview of a whole representative transverse section through the gland space is shown. Insets: magnified views of a dorsal (top) and a ventral (bottom) mucus gland; black arrowheads: positive staining, white arrowheads: equivocal staining, Scale bar = 25 µm; CRO Crossmons’s light green trichrome including Mayer’s haematoxylin and Alcian blue, OIO Oil Red O, PAS Periodic acid-Schiff, PAS-D Periodic acid-Schiff-Diastase, AB Alcian blue (pH =1, pH =2.5), NIN Ninhydrin-Schiff, COO Coomassie blue, BRO Mercuric bromophenol blue (pH =3.8, pH =4.6). CH Chromatophore, CO Collagen, DE Dermis, DP Digital phalanx, DU Mucus gland duct, ED Epidermis, MG mucus glands. OIO- and NIN-stained sections were imaged using differential interference contrast

Crossmon trichrome staining in combination with Mayer’s haematoxylin and Alcian blue (CRO; Fig. 3, Additional file 1: Table SI.4) reveals that the walls of the dorsal and ventral digital mucus glands consist of single layers of columnar mucocytes with basal nuclei. The apical cell portions are filled with cytoplasm and flocculent content. Locally, mucosubstance is observed within single mucocytes. The gland lumina are largely empty, but occasional turquoise staining of flocculent content (by Alcian blue) confirms the presence of mucosubstances. Staining intensity is particularly high for the material in the ventral mucus ducts, suggesting local concentrations of mucus.

Periodic acid-Schiff staining (PAS; Fig. 3) results in violet staining of the mucosubstance described above. Similar observations are made for PAS staining following diastase treatment (PAS-D), hence excluding glycogen as sole source of positive PAS staining. The staining intensity of the mucosubstance is pH-dependent for Alcian blue: Whereas at pH =1 (AB-1; Fig. 3) no or only faint blue staining is observed, at pH =2.5 (AB-2.5) the mucosubstance in the lumen and in the mucocytes stains clearly turquoise.

All four protein stains resulted in negative or equivocal staining. Ninhydrin-Schiff staining (NIN; Fig. 3) generally is faint for the mucus glands and their content, if compared for example to the ventral collagen layer or epidermal tissues. Also for Coomassie blue (COO; Fig. 3), the mucocytes and their secretory products stain only weakly compared to the ventral collagen and epidermal tissue. However, COO clearly accentuates the outlines of the glands. In contrast to NIN and COO, mercuric bromophenol blue (BRO; Fig. 3) causes strong, seemingly unselective staining of epidermal, dermal, and glandular tissues (greenish-blueish staining at pH =3.8, BRO-3.8; blue staining at pH =4.6, BRO-4.6). The flocculent mucosubstance stained by PAS and AB is not stained by BRO.

Finally, lipids were observed using Oil Red O (OIO; Fig. 3) only in the medullary cavity of the terminal phalanx and in a thin layer basally of the dorsal epidermis that coincides with the distribution of cutaneous melanophores.

### Mucus chemistry

#### Bulk chemistry

ATR-IR spectroscopy allows the investigation of small amounts of mucus residues adsorbed to a silicon crystal due to a multiple bounce geometry. With a typical probe depth of ∼250 nm, the signatures observed in the obtained ATR-IR-spectra reflect the bulk composition of the mucus.

**Fig. 4:**
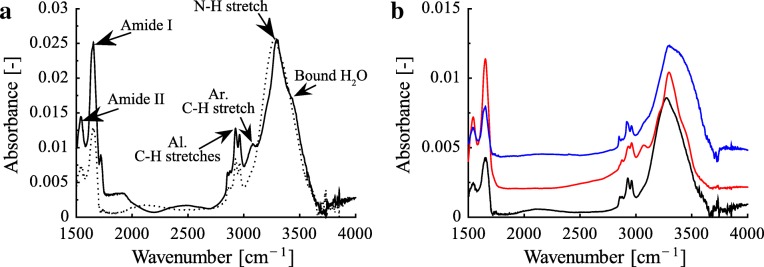
ATR-IR spectra of frog mucus. **a** Comparison of ventral digital (solid line) and abdominal (dotted line; absorbance scaled by ×3) mucus in *Hyla cinerea*. Vibrational peaks attributed to amide, N-H stretch, aromatic C-H stretch, and aliphatic C-H stretches were obtained. **b** Comparison of abdominal mucus spectra of the arboreal frog species *H. cinerea* (black) and *Osteopilus septentrionalis* (red; absorbance scaled by ×0.1) as well as the terrestrial species *Pyxicephalus adspersus* (blue; absorbance scaled by ×0.5). The spectra are offset along the ordinate axis in steps of 0.002 for clarity

The ATR-IR spectrum collected for the ventral digital mucus in *H. cinerea* shows amide I (∼1640 cm^-1^), amide II (∼1540 cm^-1^), and N-H stretch (∼3280 cm^-1^) peaks [36, 37] as well as a shoulder peak (∼3060 cm^-1^) assigned to aromatic C-H stretch vibrations, which indicate a proteinaceous component of the mucus (Fig. 4a). Further, the presence of aliphatic C-H stretch peaks (symmetric and asymmetric methylene and methyl stretches) in the hydrocarbon region (2800–3000 cm^-1^), along with a shoulder around 3500 cm^-1^ assigned to OH vibrations, indicates carbohydrates, consistent with our histochemistry results. A similar spectral profile is observed for the ventral abdominal mucus. However, the relative composition of the proteinaceous mucus components could vary between the two as seen in the stronger N-H peak relative to hydrocarbon peak for the digital mucus compared to abdominal mucus.

To evaluate the similarity in chemistry between digital and abdominal mucus, we compared the peak area ratios of the amide I and amide II peaks of the ventral digital (1.52 ±0.40, *n*=4) and abdominal (0.99 ±0.03, *n*=2) mucus in *H. cinerea*, as this ratio is sensitive to the protein structure [37]. However, no significant differences are found between the digital and abdominal median peak area ratios of the amide I and amide II peaks in *H. cinerea* (Wilcoxon rank sum test, ranksum =7,*p*=0.20) and *O. septentrionalis* (ranksum =18,*p*=0.13, Additional file 1: Figure SI.4), confirming a similarity in the chemical nature of the digital and abdominal mucus in both tree frog species.

Finally, comparison of the ATR-IR spectra of the ventral abdominal mucus across the arboreal frog species *H. cinerea* and *O. septentrionalis* as well as the terrestrial species *P. adspersus* suggests that the mucus chemistry is similar in all tested species (Fig. 4b and Additional file 1: Figure SI.4).

#### Surface chemistry

Residues of (digital or abdominal) mucus on a sapphire substrate were investigated using SFG. SFG has a probe depth of only a few nanometers, making it a highly surface-sensitive tool for monitoring interfacially ordered molecular groups.

**Fig. 5:**
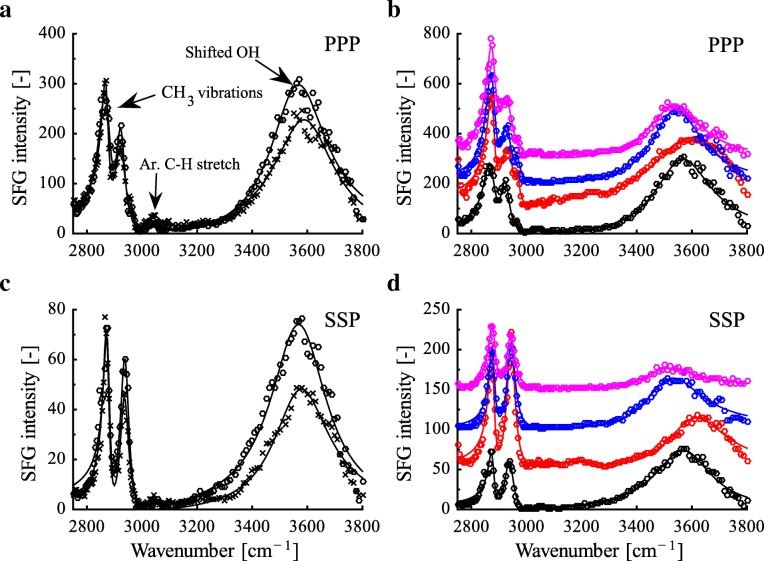
SFG spectra of frog mucus. **a** Comparison of ventral digital (crosses) and abdominal (circles) mucus in *Hyla cinerea* in (**a**) PPP and (**c**) SSP polarisations. CH_3_ vibrations include methyl symmetric, methyl Fermi, and methyl asymmetric vibrations. **b** Comparison of the ventral abdominal mucuses of the arboreal frog species *H. cinerea* (black) and *Osteopilus septentrionalis* (red) as well as the terrestrial species *Pyxicephalus adspersus* (blue) and *Ceratophrys cranwelli* (magenta) in (**b**) PPP and (**d**) SSP polarisations. The solid lines represent the fitting curves using the Lorentzian equation (Eq. 2). The spectra are offset along the ordinate axis in steps of 100 (PPP) and 50 (SSP) for clarity

In the hydrocarbon region (2750–3100 cm^-1^), we observed methyl symmetric (2875 cm^-1^), methyl Fermi (2940 cm^-1^), and methyl asymmetric (2955 cm^-1^) vibrations in the PPP polarization spectrum for the digital mucus of *H. cinerea* (Fig. 5; [38]). Similar results were obtained for SSP polarization, where only methyl symmetric and methyl Fermi vibrations were observed (Fig. 5). The presence of predominantly methyl vibrations at the interface in both PPP and SSP polarization may be associated to the presence of a well-ordered monolayer formed by long chain fatty acids or lipids present in the mucus [39, 40]. Additionally, a small peak at ∼3050 cm^-1^ assigned to the C-H stretch of aromatic amino acids groups [38, 41, 42] indicates hydrophobic amino acid residues. The hydroxyl region (3100–3800 cm^-1^) shows a broad peak around ∼3550 cm^-1^ in both PPP and SSP polarizations. This peak is caused by strong polar (typically acid-base) interactions between molecules present in the ventral digital mucus and sapphire surface hydroxyls, causing the shift of a typically sharp peak at ∼3710 cm^-1^ (assigned to the O-H stretch vibrations of the sapphire free surface hydroxyl groups [43]) towards lower wavenumbers.

Similar to the ventral digital mucus, the SFG spectra for abdominal mucus show peaks in the hydrocarbon region and the hydroxyl region associated with lipid-like mucus constituents and polar interactions of the mucus (Fig. 5a, c). The interaction strength of the abdominal mucus was compared to that of the digital mucus using the average peak shift of the sapphire surface hydroxyl peak. The average sapphire hydroxyl peak shifts for abdominal and digital mucus were 117±14 cm^-1^ (*n*=2) and 94±23 cm^-1^ (*n*=3) in PPP polarization, and 116±2 cm^-1^ (*n*=2) and 109±6 cm^-1^ (*n*=3) in SSP polarization, respectively (Additional file 1: Figure SI.5). However, no significant differences were observed between the mucuses from two different body locations (ranksum =7,*p*=0.40 for PPP; ranksum =6.5,*p*=0.40 for SSP), suggesting the conservation of the mucus surface chemistry across different body parts. In *O. septentrionalis*, also no significant differences were observed between the digital and abdominal mucus (ranksum =14.5,*p*=0.20 for PPP; ranksum =15,*p*=0.10 for SSP; Additional file 1: Figure SI.5).

Further comparisons were made across the abdominal mucus collected from the four different frog species (Fig. 5b, d). The spectral signatures (methyl vibrations with a shifted sapphire hydroxyl peak) observed for *O. septentrionalis*, *C. cranwelli*, and *P. adspersus* are similar to *H. cinerea*, indicating no or only little changes of the mucus surface chemistry in the evolution of anurans.

## Discussion

This study offers a comparative analysis of the mucus glands and their secretory products in the digital pads of tree frogs, in order to evaluate the function(s) of gland morphology and mucus chemistry related to the attachment of these animals. We will first discuss the morphology of the digital mucus glands in tree frogs, before addressing the chemistry of the secreted mucus.

### Mucus gland morphology

In all anurans, mucus glands cover large fractions of the body surface and seem to be the most uniform gland type [44]. Surprisingly, the mucus glands and their secretory products have received relatively little attention in previous research. Our 3D analysis of the mucus glands of anurans—and of the digital glands of tree frogs in particular—shows that the digital glands vary in shape and size, allowing the distinction of dorsal and ventral glands based on features other than gland location.

The morphology of the dorsal glands agrees with general descriptions for amphibian mucus glands [14, 15, 17, 19, 45, 46]. The diameter of the dorsal digital glands of 45.0 ±5.5 µm measured in our study falls in the range of values reported for mucus glands in the dorsal skin of other anurans (34.8 − 47.9 µm [47]; 42.0 − 94.9 µm [46]; 24.0 − 76.8 µm [45]; 41.6 − 52.2 µm [47]; 47.0 − 95.0 µm [48]; 46.2 − 63.0 µm [49]; 19.2 − 225.6 µm [50]). The dorsal pore density of ca. 190 mm^-2^ lies at the high end of the range of previously measured values for various anuran families (32–128 mm^-2^ [51]; 60 mm^-2^ [52]; 30–50 mm^-2^ [53]; 8–190 mm^-2^ [54]; 55–253 mm^-2^ [48]; 17–76 mm^-2^ [50]). Ecomorphological studies are needed to investigate if variations in gland density relate to the habitat, with higher gland densities in species living in arid areas [48].

To our knowledge, the volume of the mucus glands has not been measured directly before. Compared to earlier estimates (0.1 nL [51]; 1 nL [55]), we measured lower volumes for the dorsal glands (0.05 nL), despite including the mucus duct in the volume. The volumes measured in this study may be underestimated due to potential pad shrinkage during sample preparation. The discrepancy between the gland volumes and densities measured here and the ones reported in literature may be explained by our 3D-analysis, which allows the identification and accurate quantification of all glands in a given sample. We conclude that the dorsal digital mucus glands of *H. cinerea* have a similar geometry as the mucus glands of other body parts and species, except for the distinct ventral digital glands discussed below.

### Digital mucus gland clusters in anurans

The morphology of the ventral digital mucus glands deviates strongly from the general morphology of the ‘regular’ mucus glands found in the dorsal dermis: The glands are clustered in the proximal part of the subdermal pad cavity rather than distributed across the stratum spongiosum, the gland bodies are elongated instead of spherelike, and the ventral glands are significantly larger than the dorsal ones (Fig. 1 and Additional file 1: Figure SI.1). This difference in size between dorsal and ventral digital glands in *H. cinerea* was also observed, but not quantified, previously [29]. The elongated shape and the subdermal clustering of the ventral glands were both previously described in *H. cinerea* [34], *H. arborea* [24, 26–28, 56], and *Rana temporaria* [25], although not always together in a given species. The ventral digital mucus glands may represent a ‘modified form’ of the dorsal ones [56].

Although not explicitly described in the according references, screening the available literature reveals the presence of digital mucus gland clustering in numerous anuran groups other than *Hyla* (Additional file 1: Table SI.5). This shows that a cluster of digital mucus glands is not unique to *H. cinerea* or hylids, but is present in at least 10 neobatrachian families, although sometimes described as rows instead of clusters [27, 57].

Locally clustered amphibian glands are also termed macroglands [17]. In amphibians, several granular macroglands have been described, which generally are involved in defence, reproduction, and communication [14, 44]. We identified an anuran mucus macrogland—namely the subdermal cluster of digital mucus glands—in the digital pads of tree frogs and other neobatrachian families. As for the already known granular macroglands [14, 17, 44, 58], the ‘strategic’ positioning of the digital gland cluster suggests a specific functionality. However, the digital macrogland is not only present in arboreal species, but also in terrestrial ones [27].

### Functional morphology of the digital gland cluster

The presence of the ventral mucus gland cluster also in not purely arboreal species (e.g. *Acris gryllus* or *Rana temporaria*; Additional file 1: Table SI.5) supports the hypothesis that the morphology of the cluster is determined by generic functional requirements arising from ‘ground contact’, and possibly by specific requirements for climbing and attachment.

Repeated substrate-contact during locomotion leads to a loss of mucus from the ventral pad surface to the environment. This loss presumably is enhanced by the high wettability of the mucus, as indicated by the lipid-like substances found in our study, which potentially act as surfactants, and by measured contact angles <10^∘^ [59]. Compared to the dorsal pad side, we showed for the tree frog *H. cinerea* an approximately 2.4 higher total gland volume at the ventral side. Hence, mucus loss is compensated by enhancing the volume of individual glands rather than by increasing the number of glands. Enlarging or multiplying ‘regular’ glands may reduce the mechanical strength of the ventral cutis, which is an important mechanical link between the adhesive pad surface and the internal skeleton [30]. Thus, the location of the ventral glands in the internal pad space enables a 4.3 times higher volume per pad surface area, compared to the dorsal pad surface, helping to avoid desiccation of the ventral pad surface. After secretion, mucus may be stored in the hierarchical network of micro- to nanoscopic channels formed between the ventral epidermal surface cells [60]. Such mucus storage may be especially important to reduce the metabolic costs of mucus production as frogs cannot suck mucus back into the ducts [52].

A sufficient mucus volume is also important for attachment. Capillary adhesion relies on the formation of a liquid bridge between digital pad and substrate, and the question remains if tree frogs possess enough mucus to form this bridge themselves, or if an accumulation of environmental water is necessary [61]. The minimal volume of the liquid bridge can be approximated with a geometrical model of the pad-substrate gap [23]. For a circular ventral pad surface of 1.1 mm^2^ (measured in this study) and a median pad-substrate gap width of 6 nm [22], the model predicts a volume of the liquid bridge of 2.3 nL. For the axisymmetric volume of the free meniscus, we estimate a volume of the same order of magnitude. With a total ventral gland volume of around 38 nL, tree frogs seem to be well able to create capillary adhesion using only secreted mucus, assuming that the whole ventral gland volume is simultaneously available at the pad surface.

The volume enhancement by larger and longer, but fewer ventral glands may be explained by hydrodynamic considerations. The mucus flow through the glandular ducts during secretion can be approximated as steady pipe flow, described by the Hagen-Poiseuille equation:

1 $$\begin{array}{@{}rcl@{}}  \Phi = \frac{2 \pi d^{4} \Delta p}{\mu L}, \end{array} $$

where *Φ* is the volume flux generated by a pressure difference *Δp* driving a liquid with a dynamic viscosity *μ* through a circular pipe with length *L* and diameter *d*. While moving the glands into the inner pad space inevitably increases *L* and reduces the volume flux, the enlargement of the ventral glands (and hence of *d*) has an opposite, stronger, effect. The same mucus flux as in the dorsal glands can be obtained in the ca. 20-fold longer ventral glands with a duct diameter that is approximately doubled.

Further potential adaptations to counteract a reduction in volume flux with increasing duct length include changes to the mucus chemistry (i.e. the viscosity *μ*; see Eq. 1). For *L. caerulea*, a mucus viscosity of 1.43 mPas has been measured [22], which is only ca. 50% higher than the viscosity of water at room temperature and arguably almost as low as possible in an animal, potentially resulting in a low work needed for secretion.

The glandular secretion mechanism plays a major role in generating the pressure difference driving mucus flow. Generally, amphibian mucus glands secrete their content by sympathetic autonomic innervation of the myoepithelial cells—sometimes also referred to as smooth muscle fibres [62, 63]—surrounding the glands [64], leading to continuous [44] and synchronous [65, 66] mucus secretion. In the closed pad-substrate gap, continuous secretion is not needed, and may even enhance the loss of mucus. Moreover, the long ventral gland ducts cause a higher viscous resistance against secretion. Accordingly, it may be expected that the secretory mechanism differs between the ventral and dorsal glands. Contraction of the glandular muscle fibres previously was assumed to be the primary driver of digital mucus secretion [34]. However, our study does not show a stronger myoepithelial support of the ventral glands (i.e. an increased number or thickness of the myoepithelial cells covering the ventral glands compared to the dorsal ones; Additional file 1: Figure SI.3), disagreeing with the hypothesis of the glandular muscle fibres driving secretion. An analysis of the arrangement of the myoepithelial structures surrounding the ventral digital mucus glands—as performed for the salamander *Tylototriton verrucosus* [63]—may illuminate their biomechanical relevance.

A secretory mechanism independent of myoepithelial action may be found in the internal clustering of the ventral glands below the distal phalanx. Flexion of the distal phalanx by contraction of the flexor muscles [67] would help to build up pressure within the glands, effectively enhancing *Δp*. The location of the glands in the internal pad space also helps to avoid unintentional mucus secretion by mechanical loading of the ventral pad cutis during locomotion.

Finally, an adhesive function of the ventral digital glands (and their secretions) is indicated by the spatial distribution of the mucus pores. We showed that the pores are distributed across the whole ventral pad surface, in contrast to earlier observations of the ducts only ending in the cuticular groove around the digital pad [26].

However, the density of the pores, inversely approximated by their nearest-neighbour-distance, increases by 40% from the distal to the proximal side of the pad surface (Fig. 2). This suggests more mucus being present proximally than distally, which is also shown by the quadratic increase in the cumulative gland volume from distal to proximal (Additional file 1: Figure SI.2). During attachment, tree frogs regularly pull the digital pads towards the body, resulting in proximally directed sliding [28, 68] across the mucus secreted by the proximal mucus glands, which presumably distributes the mucus over the ventral pad surface. Such an evenly distributed, relatively thick mucus layer could support detachment by weakening vdW, capillary, or hydrodynamic attachment forces.

### Mucus chemistry

We used histochemical and spectroscopic methods in our chemical analysis of the digital mucus in tree frogs, which is needed to understand its role in attachment [69].

#### The chemical composition of tree frog mucus

Overall, neither the histochemical nor the spectroscopic (ATR-IR and SFG) analyses reveal major variations in the types of molecules (i.e. mucosubstances, proteins, and lipids) and in the spectroscopic characteristics of these molecules present in frog mucus as a function of location on the body (ventral digital, dorsal digital, or ventral abdominal) or lifestyle (arboreal or terrestrial). Whereas variations on an intramolecular level, such as the presence of specific functional groups, may have not been picked up by histochemistry, spectroscopic analyses do not produce evidence for such variations.

The histochemical examination of the digital mucus of *H. cinerea* reveals carbohydrate components such as neutral polysaccharides, glycoproteins, and -lipids (indicated by Periodic acid-Schiff), as well as acidic (i.e. carboxylated) but no sulfated mucopolysaccharides (indicated by Alcian blue). These results agree with the majority of histochemical analyses of anuran mucus glands (Additional file 1: Table SI.5), including the digital glands of hylids [29, 70]. Acid mucopolysaccharides have been indicated in *H. cinerea* also by positive staining with toluidine blue [29]. The flocculent nature of the mucosubstances has been described previously [15, 71], confirming that the mucus was not washed out prior to or during the staining. The good agreement of our findings on samples of *H. cinerea* that were not fixed directly after the death of the animals with literature results on immediately fixed samples (Additional file 1: Table SI.4) suggests that *post mortem* changes in mucus chemistry were insignificant. The intraglandular variations in staining intensity observed in this study have also been described in *H. cinerea* [29] and *Rana fuscigula* [69], with individual mucocytes reacting differently to PAS and AB staining. Such differences may be explained by different biosynthetical stages of single mucocytes [15, 45, 69].

All protein-specific stainings generated negative results. Mercuric bromophenol blue has been used repeatedly as ‘general protein stain’ ([58, 72]; Additional file 1: Table SI.4), but its specificity is not fully resolved [73, 74]. The weaker staining by BRO of the glands compared to other tissues can be interpreted as a negative result [58]. Arguably, a single protein staining would not be conclusive, but the combination of negative results from three different tests is evidence for the absence of proteins in the digital mucus of tree frogs ([75]; or concentrations too low to be detected by histochemistry). Negative staining of non-digital anuran mucus glands with Ninhydrin-Schiff, Coomassie blue, and Bromophenol blue was also found in other studies using various fixation treatments (Additional file 1: Table SI.4). In contrast, the signatures observed in the ATR-IR spectra indicate a protein-like constituent of digital and abdominal mucus. Proteins could be present by themselves (in small quantities) or as part of the histochemically identified glycoproteins. Also in the SFG spectra of both digital and abdominal mucus, the strongly ordered methyl peaks (in both PPP and SSP polarizations) may be explained by the presence of molecules containing hydrophobic amino acids with their side chain methyl groups ordered at the air interface. The absence of a N-H peak (typically at 3300 cm^-1^; [76]) could be due to a disordered protein backbone. Interestingly, indications of methyl and methylene groups as found in the amino acid side chains in mucosubstances were also observed in the SFG spectrum of the tongue mucus of the frog *Ceratophrys* sp. [42]. A future analysis of IR spectra below 1500 cm^-1^ and of SFG spectra in the amide I/II region would allow a direct comparison between the two spectroscopic methods and hence an investigation of the proteinaceous components of tree frog mucus (e.g. the confirmation of glycoproteins), which was not possible here due to technical limitations of our setups.

We did not detect lipids or lipoproteins in the mucus using Oil Red O (OIO) staining. Importantly, we found lipids inside the medullary cavity and in a thin subepidermal layer, which has both been observed previously [77, 78], confirming functioning of our OIO protocol. In contrast to histochemistry, the strongly ordered methyl peaks in the SFG spectra for the digital and abdominal mucus could reflect the presence of a well-ordered self-assembled monolayer formed by long chain fatty-acid-like molecules (or lipids). These opposed findings may be due to low concentrations of lipids present in the mucus along with a difference in sensitivity between the used histochemical and spectroscopic techniques. Literature reports on lipids in frog mucus are inconclusive. The presence of lipids has been rejected by various stains in other hylids [46, 47, 79], bufonids [80], and pipids ([75]; Additional file 1: Table SI.4). Using other chemoanalytical methods, however, several authors reported lipids in frog mucus [28, 56, 79, 81]. Many of these lipids are commonly found in plants [79], possibly hinting towards sample contamination. In our study, the similarity of the ATR-IR and SFG spectra between the tested arboreal (kept without plants) and terrestrial (kept with plants) species suggests that such contamination did not occur. In conclusion, the presence of lipids in digital tree frog mucus is still uncertain.

Overall, our results indicate that the mucus chemistry has been largely conserved in frogs. This suggests that the digital mucus of tree frogs (a) fulfills generic functions of amphibian mucus, and (b) possibly got incorporated into the attachment apparatus without extensive modifications of its chemistry. Detailed analyses of the mucus molecules using mass-spectrometry and of the concentration of these molecules are required to advance the understanding of the functionality of frog mucus and the mucus of tree frogs in particular. In such studies, tree frog mucus has to be considered a heterogeneous substance that originates from mucocytes and glands of varying biosynthetical stages [45, 69], epidermal transudate secretion [19, 82], and environmental liquids [19].

#### Functional chemistry of digital tree frog mucus

The chemical similarity of the dorsal and ventral digital mucus in tree frogs suggests that the mucus at the contact interface fulfills the same functions as regular amphibian mucus. These functions comprise, most importantly, cutaneous respiration, homeostasis, water regulation, defence, and lubrication [16–19, 83].

Proteoglycans such as the mucopolysaccharides detected here by PAS- and AB-staining are affine to water [84], enabling the formation of a mucus layer lubricating the skin [18, 84]. The resulting reduced skin friction makes it more difficult for predators to grip the frog [18], and reduces abrasive wear and damage of the soft skin [16], as indicated by the proximal concentration of mucus on the ventral pad surface found in our study. Similarly, in the burrowing caecilian *Siphonops annulatus* mucus glands are concentrated anteriorly [85]. In the digital pads of tree frogs, however, a reduced friction would be detrimental as the push-off force parallel to the substrate—for example during jumping—would be lowered, too. Here, the epidermal nano-/micropatterns found on the pad surface may help to drain excess liquid, hence increasing friction as well as supporting the potentially involved mechanisms of ‘dry’ and ‘wet’ adhesion by reducing the pad-substrate gap width [23, 60, 86].

The presence of large molecules, such as the polysaccharides and glycoproteins detected here, suggests an increased viscosity of the mucus compared to pure water [18, 84], which affects the generation of hydrodynamic adhesive forces. These forces scale with the concentration of mucosubstances in water [68], assuming a constant distance between pad and substrate. Although not believed to play an important role in tree frog attachment [23], a quantification of the mucus viscosity is required for an investigation of its role in the generation of hydrodynamic attachment forces. Dilution of the mucus by environmental water (e.g. rain), and non-Newtonian liquid behaviour due to the presence of mucosubstances—potentially as a function of the concentration of these substances—should be considered in such analyses. Frogs may modulate the concentration of mucosubstances to control the relative contribution of hydrodynamic and capillary adhesion (as shown in a technical system; [87]), depending on the circumstances.

The mucus chemistry could also play a role in the capillary adhesion of tree frogs. Generally, capillary forces scale with the surface tension of the liquid forming the meniscus, and with the area covered by the liquid bridge [88]. Our ATR-IR and SFG results indicate the presence of amphiphilic lipid-like molecules. Similar to surfactants, such molecules help in reducing the effective surface tension and enhancing the wetting of a liquid. The presence of surfactants in tree frog mucus is also indicated by the low contact angles of tree frog mucus measured on hydrophilic and hydrophobic substrates [59]. Similar to the balance between spreading and viscous resistance observed in spider glue [89], a trade-off between wetting (i.e. contact area) enhancement and surface tension reduction as a function of surfactant concentration may be present. In a technical adhesive system [90], the concentration of surfactants in a capillary bridge determines the stability of the bridge, and as a result the amount of adhesive forces. Analogously, tree frogs could control their attachment strength by varying the concentration of lipids in the mucus. In fact, complete failure in attachment of tree frogs has been observed when adding artificial surfactants such as soap or detergent into the pad-substrate gap [91]. Such attachment failure may be caused by the loss of van der Waals (vdW) forces due to formation of a stable mucus layer in the pad-substrate gap [90], and of capillary adhesion due to collapse of the capillary meniscus. Further studies on surfactant-mediated mechanisms of attachment failure are needed.

The evolution of anurans may have led to a mucus composition that exploits the aforementioned trade-offs while minimising the metabolic costs of the production of the required mucus components. To further explore these trade-offs, dynamic measurements of the mucus composition and of the generated attachment forces are required. Also, a more elaborate analysis of the protein content of tree frog mucus, as outlined by Hennebert et al. [92], may help to understand the role of these potentially adhesive molecules in attachment.

## Conclusions

Despite a variety of functions related to physiological processes and to attachment, anuran mucus glands and their secretions have received relatively little attention in previous research. To our knowledge, we present here the first 3D, quantitative assessment of anuran mucus glands in the adhesive digital pads of the tree frog *H. cinerea*. We show that the ventral digital mucus glands form a digital gland cluster, or macrogland, with a 2.4-fold larger volume of available mucus compared to the ‘regular’ dorsal glands, which presumably helps to compensate mucus loss while reducing unintentional mucus secretion and structural weakening of the adhesive epidermis. The gland cluster is also present in non-climbing frog families and hence may represent a previously unrecognised adaptation of amphibians to a terrestrial lifestyle. Using histochemical and spectroscopic methods, we found indications of carbohydrates, proteinaceous, and lipid-like substances in frog mucus, and show that frog mucus varies only little between different body locations (digital vs. abdominal) and as a function of lifestyle (arboreal vs. terrestrial). This result indicates a conservation of the cutaneous mucus chemistry in the evolution of anurans. Our functional and comparative analysis of the digital macrogland morphology and mucus chemistry contributes to a better understanding of the bioadhesion of these animals, and of anuran evolution. As the ventral digital mucus and cutis are the primary barrier against environmental pathogens, further studies on these components may also help to understand the current global decrease in amphibian populations due to the fungus *Batrachochytrium dendrobatidis* [93]. Finally, highlighting analogies between the glandular systems of tree frogs and of other attachment systems in nature (e.g. in insects), and placing these analogies in a functional context, will advance the general understanding of bioadhesion and may stimulate novel trends in the design of bioinspired adhesives.

## Methods

### Experimental animals

For histochemical and morphological analyses, we used three adult *Hyla cinerea* that died of unknown causes (*post mortem* snout-vent-length *ℓ*_SV_= 40–46 mm, body mass *m*= 6.2–8.2 g, age ≤1 year). For a description of the housing conditions, see [30]. We collected the distal limbs before 5:30 h after death by disarticulation of the elbow and knee joints. Until further use, the right hindlimb of each individual was quick-frozen in liquid nitrogen and subsequently stored at −80^∘^. All following steps were executed at −20^∘^, unless mentioned otherwise. For µ-CT, we chemically fixated and stored one digit of the left hindlimb as described elsewhere [30].

For comparative spectroscopic analyses of the mucus chemistry, two adult *H. cinerea* (*ℓ*_SV_= 40–44 mm, *m*= 2.1–4.2 g) and four adult *O. septentrionalis* (*ℓ*_SV_= 69–85 mm, *m*= 14.7–28.1 g) were acquired from commercial vendors and housed individually in plastic containers with water *ad libitum* and biweekly feedings of gut-loaded cockroaches. Three juvenile individuals each of *C. cranwelli* (*ℓ*_SV_= 32–45 mm, *m*= 4.7–9.7 g) and *P. adspersus* (*ℓ*_SV_= 32–37 g, *m*= 2.1–3.9 g) were acquired as part of an unrelated study and mucus samples were taken before euthanasia; both species are terrestrial ambush predators.

### 3D reconstruction of the digital gland morphology

We analysed the morphology of the digital glands in *H. cinerea* in 3D using µ-CT. The digit used for µ-CT was contrast-stained, scanned, and segmented as described in [30]. We segmented all glands opening to the ventral pad surface (termed ventral glands) and—to reduce the efforts of manual segmentation—the left half of the glands opening to the dorsal pad surface (termed dorsal glands).

We quantified the morphology of the individual glands using a custom-made MATLAB routine (Version R2016b, The Mathworks, USA). Voxel size was set to 1.3×1.3×1.3 µm^3^ (original µ-CT voxel size: 0.65×0.65×0.65 µm^3^) to reduce the computational effort. Individual volumes of dorsal and ventral glands were computed by summing up the segmented voxels. Using MATLAB, the dorsal (*V*_dg_) and ventral (*V*_vg_) gland volumes were tested for normal distribution using an one-sample Kolmogorov-Smirnov test, and for a difference of the means of the two groups using a two-sample t-test. We used for all statistical analyses a significance level *α* = 5%. Further, each ventral gland was skeletonised using an accurate fast marching algorithm to extract the length of the gland centerline *l*. The distal end of the centerline was used as the spatial coordinate of the mucus pore. The location of the dorsal pores was set as the most dorsal segmented voxel of each gland. Pore density was approximated by the nearest-neighbour-distance (NND) between the pores. To test for spatial patterns in the distribution of the mucus pores, we performed with MATLAB a multiple linear regression of NND as a function of the lateral and longitudinal pore coordinates (*x* and *y*), using t-tests to identify significant deviations of the fitted slopes from 0. The dorsal and ventral pad surface area was determined by Delaunay triangulation of the respective pore vertices, and by taking the sum of the resulting triangular areas.

### General chemistry of glandular mucus

We applied various cryo-histochemical stains to investigate which classes of molecules are present in tree frog mucus. The staining of the mucus still present in the glands helps to reduce contamination with, for example, environmental substances, mucus from other body parts, or non-mucus related molecules such as cuticular proteins or lipids. Before histological staining, the two most distal segments of digits of the hindlimb of *H. cinerea* were cut from the frozen limb through the central part of the middle phalanx. The frozen samples were mounted on specimen discs using KP-CryoCompound (Klinipath, The Netherlands), cut perpendicularly to the longitudinal digital axis (i.e. transverse) into 7 µm thick sections (10 µm for Ninhydrin-Schiff staining) using a CM3050S cryostat (Leica Microsystems B.V, The Netherlands), and placed on object slides (Menzel, Germany). The good agreement of our histological results with literature reports on freshly killed animals as well as with our spectroscopic results suggests that changes in the mucus chemistry due to *post mortem* decay were negligible.

As general overview stain, we applied Crossmon’s light green trichrome stain (CRO) in combination with Mayer’s haematoxylin and Alcian blue as described in [30]. We used Periodic acid-Schiff stain (PAS) according to Romeis [94, 95] to detect neutral polysaccharides, mucopolysaccharides, glycoproteins, and -lipids containing 1,2-glycols (or their amino- and alkylamino-derivatives; [96]). A diastase treatment prior to PAS-staining (PAS-D) was applied to exclude the presence of glycogen [94, 95]. To detect acid mucopolysaccharides such as sialin and uronic acids and some glycoproteins, we used Alcian blue [95]; to distinguish between highly acidic (i.e. sulfated) and acidic (i.e. carboxylated) mucopolysaccharides, Alcian blue was applied at pH =1 (AB-1) and at pH =2.5 (AB-2.5), respectively [95, 97, 98]. As general protein stains [44, 72, 75, 99], we used Ninhydrin-Schiff (NIN), which stains *α*-amino-acids by binding with their free NH_2_ groups [100–102], Coomassie blue R250 (COO, [96]), which stains proteins by a combination of hydrophobic interactions and heteropolar bonding with basic amino acids [96, 103, 104], and Mercuric bromophenol blue [73], which has been reported to react with acidic, sulphydryl, and aromatic protein residues [105]. The pH-sensitive bromophenol blue was used at pH =3.8 (BRO-3.8) and at pH =4.6 (BRO-4.6). Hydrophobic lipids (and lipoproteins; [106]) were stained with Oil Red O (OIO; [107]). By using cryo-samples and lyophobic solvents, we reduced the risk of washing out of lipid-like mucosubstances [69, 84]. Finally, Bouin-fixated sections of digits from the left hindlimb and the right forelimb were stained immunohistochemically as in [30] to test for differences in the amount of smooth muscle *α*-actin—present in muscular and myoepithelial structures—in between dorsal and ventral mucus glands. For detailed staining protocols, we refer to the supplement.

Images of the stained sections were obtained using a digital microscope camera (DFC450c, Leica, Germany) mounted on an upright microscope (DM6b, Leica) with a HC PL APO 40×/0.85 objective controlled with the Leica Application Suite X (Version 2.0). High-resolution images of whole sections were obtained by merging tile-scanned images. For relatively faint stains (NIN and OIO), differential interference contrast was used to show the internal pad structures. Post-processing (cropping, rotating, scaling, white balancing, and arranging) of the images was done in Photoshop CC (Version 2017.1.1, Adobe Systems, USA) and in Illustrator CS6 (Version 16.0.3, Adobe).

### Bulk chemistry of secreted mucus

Infrared spectra were acquired using an iS50 Fourier Transform Infrared spectroscopy (IR) system equipped with a mercury-cadmium-tellerium (MCT) detector (Thermofisher, USA). A horizontal attenuated total internal reflection accessory (PIKE Technologies, USA) was used to collect multibounce (10 bounces) attenuated total reflection infrared spectra (ATR-IR) from a pristine silicon crystal. The crystal surface was cleaned by soaking in hot Piranha solution (7:3 H_2_SO_4_ and H_2_O_2_) for 1 h followed by sonication in ultrapure water (Millipore filtration system, 18.2 M *Ω*·cm with pH = 6–7) for 1 h. Prior to cleaning, a 5 wt.% solution of hydrofluoric acid in deionized water was used to remove the native silicon oxide (SiO_2_) layer. A spectrum from the pristine crystal was used as background for the ATR-IR measurements. To obtain a spectrum, we averaged 100 interferograms with a 4 cm^-1^ step scan. ATR-IR spectra of the frog mucus were obtained by gently rubbing a clean Si crystal on the according part of the frog’s body (i.e. digital pad or abdomen) to deposit the ventral mucus on the crystal. Prior to mucus collection, the respective body part was rinsed with tap water, followed by rinsing with ultrapure water, and gently dried with a kimwipe to avoid potential contamination of the mucus from other body portions or the environment. The mucus deposit on the Si crystal surface was dried overnight in vacuum to ensure the evaporation of bound water. ATR-IR scans were then collected as described above. Calculation and analysis of peak areas were done with Igor Pro (Vers. 6.37, Wavemetrics, USA) with the Multipeak fitting analysis package (Vers. 2, Wavemetrics). At least 3 repeats from different individuals were done per species for both the digital and abdominal mucus for *H. cinerea* (2 individuals) and *O. septentrionalis* (3 individuals). One repeat was done for *P. adspersus* (1 individual), as the animal was euthanized for another experiment shortly after mucus sampling. Comparisons were made between the digital and abdominal mucus spectra for both *H. cinerea* and *O. septentrionalis*. A Wilcoxon rank sum test was performed in MATLAB to detect significant differences in the medians of the amide I/II ratios between the two species. Further comparisons were made between the IR spectra of the abdominal mucus from the three studied frog species.

### Surface chemistry of secreted mucus

SFG spectra were collected using a ps Spectra Physics laser system, details of which are provided elsewhere [39, 108]. Briefly, it involves the overlap of a fixed 800 nm visible laser beam with a tunable infrared beam (2000–3800 cm^-1^). SFG being an interface-sensitive technique, provides information about molecular stretching vibrations at the interface, where there is a breakdown in inversion symmetry. Equilateral sapphire prisms (15 cm × 15 cm × 15 cm × 10 cm, c-axis ±2^∘^ parallel to the prism face, Meller Optics Inc.) were used as substrates for the experiment. The sapphire prisms were first baked for 2 h at 760^∘^C followed by sonication with a series of different organic solvents (toluene, chloroform, acetone, and ethanol) for 45 min each. The sapphire prisms were then sonicated with ultrapure water (18.2M *Ω*·cm) for about 1 h, blow dried with N_2_, and finally plasma sterilized (Harrick Plasma, PDC-32G) for 5 min to remove remaining hydrocarbon residues. The stainless-steel sample holder was cleaned using the same method as used for the sapphire prisms with exception of the baking step. Blank scans were collected for the clean sapphire prisms using a total internal reflection geometry to ensure a clean sapphire surface and to locate the position of the sapphire free hydroxyl peak. After cleaning the frog’s body surface as described for ATIR-IR, ventral mucus was deposited by rubbing the digital pad or abdomen on the sapphire surface. At least 3 repeats (from different individuals) were done for both the digital and abdominal mucus for *H. cinerea* (2 individuals) and *O. septentrionalis* (3 individuals). One and two repeats were done for *P. adspersus* (1 individual) and *C. cranwelli* (2 individuals), respectively. The SFG spectra were collected using PPP (P-polarized SFG, P-polarized visible, P-polarized infrared) and SSP (S-polarized SFG, S-polarized visible, P-polarized infrared) polarizations, where S and P relate to the direction of the electric field with respect to the incident plane. We used the Lorentzian equation to fit the measured SFG spectra:

2 $$\begin{array}{@{}rcl@{}}  I_{\text{SFG}} \propto \left| \chi_{\text{NR}} + \sum \frac{A_{\mathrm{q}}}{\omega_{\text{IR}} - \omega_{\mathrm{q}} + i \Gamma_{\mathrm{q}}} \right|^{2}, \end{array} $$

where *χ*_NR_ describes the non-resonant contribution that does not change with scanning wavenumber *ω*_IR_. *A*_q_,*Γ*_q_, and *ω*_q_ are the amplitude strength, damping constant, and resonant frequency of the *q*th vibrational resonance, respectively.

## Additional file

Additional file 1 Supplementary material — Tree_frog_glands_and_mucus. (PDF 3030 kb)

## Abbreviations

µ-CT: Synchrotron micro-computer-tomography
AB: Alcian blue
AICc: Akaike information criterion for low sample sizes
ATR-IR: Attenuated total reflectance-infrared spectroscopy
BRO: Mercuric bromophenol blue
CH: Chromatophore
CO: Collagen
COO: Coomassie blue
CRO: Crossmon’s trichrome stain in combination with Mayer’s haematoxylin and Alcian blue
DE: Dermis
DP: Digital phalanx
DU: Mucus gland duct
ED: Epidermis
MG: Mucus gland
NIN: Ninhydrin-Schiff staining
OIO: Oil red O
PAS: Periodic acid-Schiff staining
PAS-D: Periodic acid-Schiff staining with subsequent diastase treatment
PPP: P-polarized SFG, P-polarized visible, P-polarized infrared
SFG: Sum frequency generation spectroscopy
SMA: Smooth muscle *α*-actin-antibody
SSP: S-polarized SFG, S-polarized visible, P-polarized infrared
vdW: Van der Waals
WUR: Wageningen University & Research
