# Supplementary material for: Comparative and functional analysis of the digital mucus glands and secretions of tree frogs
Source: Front Zool. 2019 Jun 13;16:19. doi: 10.1186/s12983-019-0315-z (PMC6563374; doi:10.1186/s12983-019-0315-z)
Supplement: Supplementary file 1 — Supplementary material — Tree_frog_glands_and_mucus. (PDF 3030 kb) [file 12983_2019_315_MOESM1_ESM.pdf]

SUPPLEMENTARY MATERIAL

Comparative and functional analysis of the digital mucus glands and secretions of tree frogs

Julian KA Langowski[1*], Saranshu Singla[2], Alex Nyarko[2], Henk Schipper[1], Frank T van den Berg[1], Sukhmanjot Kaur[2], Henry C Astley[3], Sander WS Gussekloo[1], Ali Dhinojwala[2] and Johan L van Leeuwen[1]

[*]Correspondence:
julian.langowski@wur.nl
[1]Experimental Zoology Group, Department of Animal Sciences, Wageningen University & Research, De Elst 1, 6708 WD Wageningen, The Netherlands
Full list of author information is available at the end of the article

Contents

1 Symbols and abbreviations **2**
1.1 List of symbols . . . 2
1.2 List of abbreviations . . . 2

2 Nomenclature of mucosubstances **3**

3 Materials **4**
3.1 Schematics of comparative approach . . . 4
3.2 Histochemistry procedures . . . 4
3.2.1 Crossmon-Alcian blue (CRO) . . . 4
3.2.2 Periodic acid-Schiff (PAS,PAS-D) . . . 5
3.2.3 Alcian blue (pH 1.0, pH 2.5; AB) . . . 6
3.2.4 Coomassie Blue R250 (COO) . . . 6
3.2.5 Mercuric bromophenol blue (BRO) . . . 7
3.2.6 Ninhydrin-Schiff (NIN) . . . 7
3.2.7 Oil Red O (OIO) . . . 8

4 Results **9**
4.1 Comparison of dorsal and ventral digital gland volumes . . . 9
4.2 Distribution of the cumulative ventral gland volume along the longitudinal pad axis . . . 9
4.3 Myoepithelial cells of dorsal and ventral digital mucus glands . . . 10
4.4 Summary of histochemistry results . . . 10
4.5 Comparison of IR and SFG results . . . 11
4.5.1 Interspecific comparison of IR spectra . . . 11
4.5.2 Interspecific comparison of hydroxyl peak shifts in SFG spectra 11

5 Discussion **12**
5.1 Histochemistry reports of anuran mucus in literature . . . 12
5.2 Anuran gland cluster reports in literature . . . 13

References **15**

1 Symbols and abbreviations

1.1 List of symbols

Table SI.1 List of symbols. a.u. arbitrary unit.

Symbol	Unit	Description
A_q	a.u.	Amplitude strength of the qth vibrational resonance
d	m	Ventral duct diameter / Pipe diameter
d_d, d_v	m	Dorsal and ventral gland diameter
l	m	Ventral gland length / length of the gland centerline
L	m	Pipe length
ℓ_SV	m	Snout-vent-length
m	kg	Body mass
n	-	Number of glands/data points
NND	m	Nearest-neighbour-distance
p	-	p-value
t	-	t-statistic
V_dg, V_vg	L	Dorsal and ventral gland volume
V_d, V_v	L	Total dorsal and ventral gland volume
α	-	Significance level
Γ_q	$\mathrm{cm^{-1}}$	Damping constant of the qth vibrational resonance
Δp	-	Pressure difference
μ	$\mathrm{kg\,m^{-1}\,s^{-1}}$	Dynamic fluid viscosity
ρ_d, ρ_v	$\mathrm{m^{-2}}$	Dorsal and ventral gland density
ρ_dV, ρ_vV	$\mathrm{L\,m^{-2}}$	Dorsal and ventral gland volume density
χ_NR	a.u.	Non-resonant susceptibility
Φ	$\mathrm{m^3\,s^{-1}}$	Volumetric flow rate, volume flux
ω_IR	$\mathrm{cm^{-1}}$	Scanning wavenumber
ω_q	$\mathrm{cm^{-1}}$	Resonant frequency of the qth vibrational resonance

1.2 List of abbreviations

Table SI.2 List of abbreviations.

Abbreviation	Description
AB	Alcian blue
AICc	Akaike information criterion for low sample sizes
ATR-IR	Attenuated total reflectance-infrared spectroscopy
BRO	Mercuric bromophenol blue
CH	Chromatophore
CO	Collagen
COO	Coomassie blue
CRO	Crossmon's trichrome stain in combination with Mayer's haematoxylin and Alcian blue
DE	Dermis
DP	Digital phalanx
DU	Mucus gland duct
ED	Epidermis
MG	Mucus gland
μ-CT	Synchrotron micro-computer-tomography
NIN	Ninhydrin-Schiff staining
OIO	Oil red O
PAS	Periodic acid-Schiff staining
PAS-D	Periodic acid-Schiff staining with subsequent diastase treatment
PPP	P-polarized SFG, P-polarized visible, P-polarized infrared
SFG	Sum frequency generation spectroscopy
SMA	Smooth muscle α-actin-antibody
SSP	S-polarized SFG, S-polarized visible, P-polarized infrared
vdW	Van der Waals
WUR	Wageningen University & Research

2 Nomenclature of mucosubstances

Throughout the manuscript, we follow in the description of the various secretory products of amphibian mucus glands largely the nomenclature proposed by Kiernan [1]:

- **Mucus** The entire secretory product.
- **Mucosubstance** Polymeric sugar-protein-complexes.
- **Polysaccharides** (also: carbohydrates) Pure oligo- or polysacharides (e.g. glycogen).
- **Proteoglycans** (also: heteroglycans) Heavily glycosilated proteins with long (hetero-)polysaccharide side-chains (e.g. mucopolysaccharides and acid mucopolysaccharides).
- **Mucopolysaccharides** (also: glycosaminoglycans, heteropolyaminosaccharides, or polyanionic glycoproteins) Proteoglycan side chains of polymeric disaccharides (e.g. Sialogycans). Acid mucopolysaccharides are also termed glycosaminoglucuronoglycans.
- **Glycoproteins** (also: mucins, i.e. glycoproteins with high sugar content) Proteins bearing covalently bound oligosaccharide chains ($2-12$ monosaccharide units).

3 Materials

3.1 Schematics of comparative approach

Table SI.3 Comparative investigation of the ventral (ven.) and dorsal (dor.) mucus glands and secretions in arboreal (A, 'tree frogs') and terrestrial (T) frogs.

Technique	Comparison	Species
Synchrotron μ-CT	ven. vs. dor. digital glands	*Hyla cinerea* (A)
Cryo-histochemistry	ven. vs. dor. digital glands/mucus	*H. cinerea* (A)
ATR-IR & SFG	ven. digital vs. ven. abdominal mucus	*H. cinerea* (A), *Osteopilus septentrionalis* (A)
	ven. abdominal mucuses	*H. cinerea* (A), *O. septentrionalis* (A), *Ceratophrys cranwelli* (T), *Pyxicephalus adspersus* (T)

3.2 Histochemistry procedures

Prior to all histochemical stainings, the cryo-sections were allowed to warm up to room temperature before fixing with 4 % formalin.

3.2.1 Crossmon-Alcian blue (CRO)

The Crossmon-Alcian blue stain was performed as described by Romeis [2]:

Solutions:

**Acetic acid** (3 %)		
Acetic acid, glacial	Merck, Germany	3 mL
Distilled water		97 mL
**Alcian blue (pH 2.5)**		
Acetic acid, glacial		3 mL
Distilled water		97 mL
Alcian Blue GS	Fluka AG, Buchs SG, Switzerland	1 g
**Mayer's Haematoxylin**		
Haematoxylin	VWR, The Netherlands	1 g
Distilled water		1000 mL
Sodium iodate	Merck, Germany	0.2 g
Potassium Alum	Merck, Germany	50 g
Chloral hydrate	VWR, The Netherlands	50 g
Citric acid	Sigma, USA	1 g
Filtrate before use		
**Fuchsin/Orange G**		
Acid fuchsin	Merck, Germany	1.3 g
Orange G	Merck, Germany	1 g
Acetic acid, glacial		5 mL
Distilled water		500 mL
Thymol	Merck, Germany	0.33 g
**Phosphotungstic acid** (5 %)		
Phosphotungstic acid	Merck, Germany	25 g
Distilled water		500 mL
**Light green** (1 %)		
Light green SF	Chroma-Gesellschaft Schmid GmbH & Co, Germany	10 g
Acetic acid, glacial		10 mL
Distilled water		1000 mL

Protocol:

1) Fixation with formalin	10 min	
2) Rinsing in distilled water	2 · 1 min	
3) Acetic acid (3%)	3 min	
4) Alcian blue (pH 2.5)	30 min	
5) Rinsing in acetic acid (3%)	20 s	
6) Rinsing in distilled water	2 · 2 min	
7) Mayer's Haematoxylin	7 min	
8) Rinsing in running tap water	10 min	
9) Fuchsin/Orange G	15 s	
10) Rinsing in distilled water	2 min	
11) Phosphotungstic acid (5%)	2 min	
12) Rinsing in distilled water	2 min	
13) Light green (1%)	2 min	
14) Rinsing in distilled water	2 · 1 min	
15) Dehydration (100% ethanol)	4 · 2 min	
16) Clearing with Xylene	3 · 2 min	VWR, The Netherlands
17) Mounting with DPX		BDH Laboratory Supplies, UK

3.2.2 Periodic acid-Schiff (PAS,PAS-D)

The Periodic acid-Schiff stain was performed as described by Romeis [2,3]:

Solutions:

**Periodic acid** (0.5 %)		
Periodic acid	Merck, Germany	2.5 g
Distilled water		500 mL
**$K_2S_2O_5$** (10 %)		
$K_2S_2O_5$	Merck, Germany	50 g
Distilled water		500 mL
**HCl (1N)**		
HCl (37%)	Merck, Germany	50 mL
Distilled water		500 mL
**SO_2-water**		
$K_2S_2O_5$ (10%)		10 mL
HCl (1N)		10 mL
Distilled water		180 mL

Protocol:

1) Fixation with formalin	10 min
2) Rinsing in distilled water	2 · 1 min
3) PAS: Distilled water / PAS-D: Saliva (diluted 1:2 with demineralised water at 37 °C)	60 min
4) Rinsing in distilled water	3 · 1 min
5) Periodic acid 0.5%	10 min
6) Rinsing in distilled water	3 min
7) Schiff's reagent	20 min
8) Freshly made SO_2-water	3 · 2 min
9) Rinsing in running tap water	5 min
10) Rinsing in distilled water	2 min
11) Counterstain with Mayer's Hematoxylin	3 min
12) Rinse in running tap water	10 min
13) Dehydration (96% to 100% ethanol)	
14) Clearing with Xylene	3 · 2 min
15) Mounting with Depex	

3.2.3 Alcian blue (pH 1.0, pH 2.5; AB)

The Alcian blue stain was performed as described by Mulisch [3]:

Solutions:

**Alcian Blue pH 2.5**		
Acetic acid 3%		
Distilled water		194 mL
Acetic acid, glacial		6 mL
Alcian Blue pH 2.5		
Alcian Blue GS		2.0 g
Acetic acid (3%)		200 mL
**Alcian Blue pH 1.0**		
0.1 N HCl		
1 N HCl		20 mL
Distilled water		180 mL
Alcian Blue pH 1.0		
Alcian Blue GS		2.0 g
0.1 N HCl		200 mL
**Kernechtrot**		
Aluminium sulphate 5%		
Aluminium sulphate $Al_2(SO_4)_3 \cdot 18\ H_2O$	Merck, Germany	19.34 g
Distilled water		200 mL
Kernechtrot		
Kernechtrot	Chroma-Gesellschaft, Germany	0.2 g
Aluminium sulphate 5%		200 mL

Protocol:

1) Fixation with formalin	10 min
2) Rinsing in distilled water	$2 \cdot 1$ min
3) HCl 0.1N (for AB pH 1.0) / Acetic acid 3% (for AB pH 2.5)	5 min
4) Alcian Blue (pH 1.0 or pH 2.5)	30 min
5) Rinsing in running tap water	10 min
6) Rinsing in distilled water	$2 \cdot 2$ min
7) Kernechtrot	5 min
8) Rinsing in running tap water	2 min
9) Rinsing in distilled water	$2 \cdot 2$ min
10) Dehydration (96% to 100% ethanol)	
11) Cleaning with Xylene	$3 \cdot 2$ min
12) Mounting with Depex	

3.2.4 Coomassie Blue R250 (COO)

The Coomassie blue stain was performed as described by Kiernan [1]:

Solutions:

**Acetic ethanol**		
Acetic acid, glacial		100 mL
Ethanol 100%	Fisher Scientific, UK	300 mL
**Staining solution**		
Coomassie blue R250	Bio-Rad, USA	40 mg
Acetic ethanol		200 mL
**Staining solution diluted 1:3**		
Staining solution		25 mL
Acetic ethanol		75 mL

Protocol:

1)	Fixation with formalin	10 min
2)	Rinsing in distilled water	2 · 1 min
3)	Ethanol 100%	10 min
4)	Diluted staining solution	15 min
5)	Acetic ethanol	5 min
6)	Dehydration (96% to 100% ethanol)	
7)	Cleaning with Xylene	3 · 2 min
8)	Mounting with DPX	

3.2.5 Mercuric bromophenol blue (BRO)

The Mercuric bromophenol blue stain was performed as described by Hornatowska [4]:

Solutions:

**Staining solution**		
Mercuric chloride	Merck, Germany	10 g
Bromophenol blue	Fluka, Switzerland	100 mg
Distilled water		100 mL
Adjust pH of staining solution (pH 3.8 and pH 4.6) with HCl/NaOH.		
NaOH	Merck, Germany	

Protocol:

1)	Fixation with formalin	10 min
2)	Hydration (Distilled water)	2 · 1 min
3)	Staining solution	15 min
4)	Acetic acid (0.5%)	2 min
5)	Rinsing in distilled water	1 min
6)	Dehydration (Tertiary butanol)	1 min
7)	Dehydration (Tertiary butanol)	2 min
8)	Clearing with Xylene	1 min
9)	Mounting with Depex	

3.2.6 Ninhydrin-Schiff (NIN)

The Ninhydrin-Schiff stain was performed as described by Bancroft [5]:

Solutions:

**Ninhydrin solution (0.5%)**		
Ninhydrin	Merck, Germany	0.5 g
Ethanol 100%		100 mL

Protocol:

1)	Fixation with formalin	10 min
2)	Rinsing in distilled water	2 · 1 min
3)	Ethanol 70%	10 min
4)	Ninhydrin solution (37 °C), freshly made	Overnight
5)	Rinsing in running tap water	3 min
6)	Schiff's reagent	75 min
7)	Rinsing in running tap water	3 min
8)	Dehydration (70% to 100% ethanol)	
9)	Clearing with Xylene	3 · 2 min
10)	Mounting with Depex	

3.2.7 Oil Red O (OIO)

The Oil Red O stain was performed as described by Lillie and Ashburn [6]:

Solutions:

Oil Red O

Stock

Oil Red O	Chroma-Gesellschaft	0.5 g
Isopropanol	Merck, Germany	100 mL

Working solution

30 mL stock + 20 mL distilled water

Allow to stand for 10 min

Filtrate and cover immediately

Protocol:

1)	Fixation with formalin	10 min	
2)	Rinsing in running tap water	2 min	
3)	Isopropanol 60%	10 s	
4)	Oil Red O, working solution, freshly made	10 min	
5)	Isopropanol 60%	1 dip	
6)	Rinsing in distilled water	$4 \cdot 1$ min	
7)	Mounting with glycerol		(Merck, Germany)

4 Results

4.1 Comparison of dorsal and ventral digital gland volumes

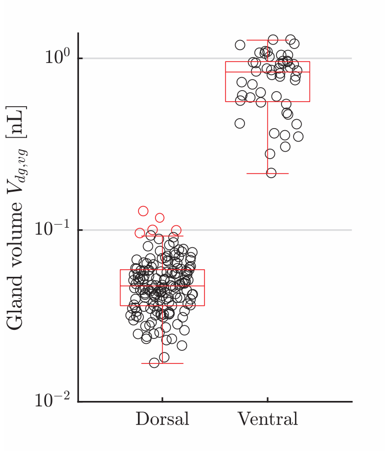

Figure SI.1 Individual digital mucus gland volumes in Hyla cinerea. Boxes indicate median, and 25th and 75th percentiles of the measured volumes of the dorsal (V_{dg}, $n = 158$) and ventral (V_{vg}, $n = 49$) digital glands. Values that are located more than 1.5 times the interquartile range above or below the boxes are shown as outliers (red circles).

4.2 Distribution of the cumulative ventral gland volume along the longitudinal pad axis

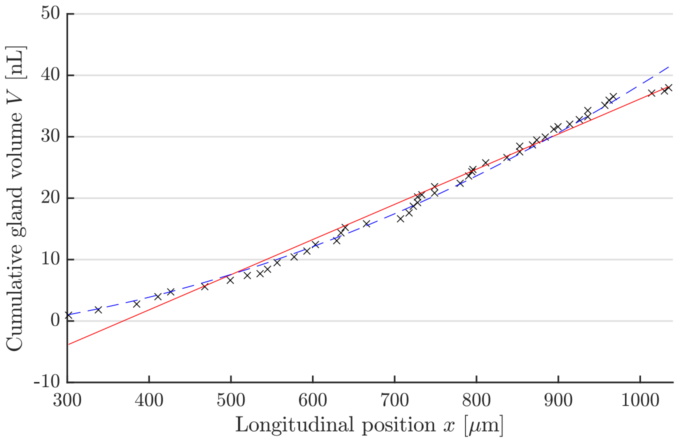

Figure SI.2 Mucus distribution on the ventral pad surface. Cumulative ventral gland volume (i.e. the summarised volume of all glands distal to a given position along the longitudinal pad axis) as a function of longitudinal position x (low x: distal, high x: proximal). For an uniform gland pore distribution, a linear curve would be expected. A quadratic model ($V = -2.7\,\mathrm{nL} + 4.1 \cdot 10^{-5}\ \mathrm{nL\,\mu m^{-2}}\ x^2$; Akaike information criterion for low sample sizes AICc $= -527.9$; blue dashed curve) fits the data better than a linear one ($V = -21.1\,\mathrm{nL} + 5.7 \cdot 10^{-2}\ \mathrm{nL\,\mu m^{-1}}\ x$; AICc $= -498.4$; red continuous curve). The quadratic trend suggests an increased mucus volume present on the proximal half of the ventral pad surface.

4.3 Myoepithelial cells of dorsal and ventral digital mucus glands

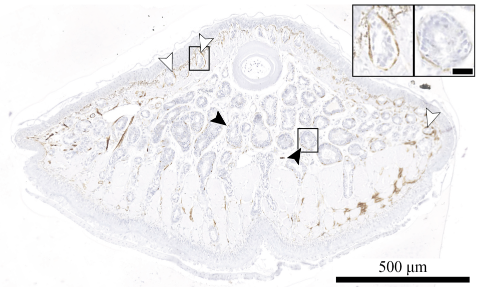

Figure SI.3 Glandular myoepithelial cells in Hyla cinerea. Transverse section of the digital pad showing the immunohistochemically stained myoepithelial cells (brown) surrounding the dorsal (white arrowheads) and ventral (black arrowheads) mucus glands. Insets: Dorsal (left) glands appear to have a more strongly developed myoepithelial sheath than ventral ones (right); scale bar $= 25\,\mu\mathrm{m}$.

4.4 Summary of histochemistry results

Table SI.4 Cryo- and immunohistochemical staining results of the dorsal and ventral mucus glands in the digital pads of *Hyla cinerea*.

		Dorsal		Ventral	
	Stain	Mucocytes	Lumen	Mucocytes	Lumen
Overview	CRO	+	+	+	+
Mucosubstances	PAS	+	+	+	+
	PAS-D	+	+	+	+
	AB-1	-/0	-/0	-/0	-/0
	AB-2.5	+	+	+	+
Proteins	NIN	-	-	-	-
	BRO-3.8	0	0	0	0
	BRO-4.6	0	0	0	0
	COO	-	-	-	-
Lipids	OIO	-	-	-	-
Muscular structures	SMA	+	N/A	+	N/A

Staining results: + Staining, - No staining, 0 Equivocal staining.
Staining method: CRO Crossmons's light green trichrome including Mayer's haematoxylin and Alcian blue, PAS Periodic acid-Schiff, PAS-D Periodic acid-Schiff-Diastase, AB Alcian blue (pH $= 1$, pH $= 2.5$), NIN Ninhydrin-Schiff, BRO Mercuric bromophenol blue (pH $= 3.8$, pH $= 4.6$), COO Coomassie blue, OIO Oil red O, SMA Smooth muscle α-actin-antibody.

4.5 Comparison of IR and SFG results

4.5.1 Interspecific comparison of IR spectra

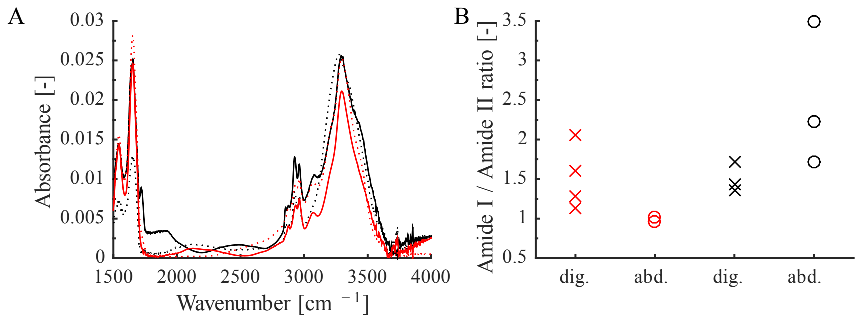

Figure SI.4 Interspecific comparison of frog mucus IR spectra.(A) Comparison of ventral digital (solid line) and abdominal (dotted line) mucus in *Hyla cinerea* (black; abdominal spectrum scaled by $\times 3$) and *Osteopilus septentrionalis* (red; digital spectrum scaled by $\times 0.8$; abdominal spectrum scaled by $\times 0.3$). (B) Comparison of amide I/amide II peak area ratios of mucus obtained from the digits (dig., crosses) and the abdomen (abd., circles) of *H. cinerea* (red) and *O. septentrionalis* (black). No significant differences were found in the peak area ratios between digital and abdominal mucus for both *H. cinerea* and *O. septentrionalis*. The statistical results are discussed in the main text.

4.5.2 Interspecific comparison of hydroxyl peak shifts in SFG spectra

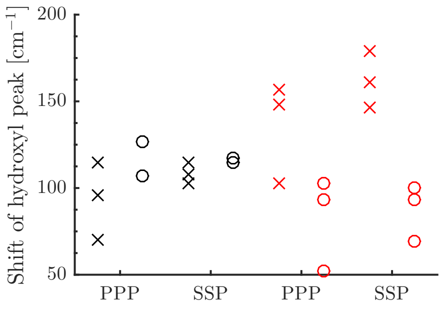

Figure SI.5 Interspecific comparison of frog mucus SFG spectra. Comparison of hydroxyl peak shifts of mucus obtained from the digits (crosses) and the abdomen (circles) of *H. cinerea* (red) and *O. septentrionalis* (black) analysed with SFG in PPP and SSP polarisation. No significant differences were found in hydroxyl peak shifts between digital and abdominal mucus for both *H. cinerea* and *O. septentrionalis* in both polarisations. The statistical results are discussed in the main text.

5 Discussion

5.1 Histochemistry reports of anuran mucus in literature

Table SI.5 Histochemical staining results for the mucus glands of neobatrachians (top) and other anurans (bottom).

Family	Species	Mucosubstances	Proteins	Lipids	Reference
Bufonidae	*Rhinella arenarum*	PAS-HE +, AB-2.5 +, HE/SAF +	COO -	SURIII -	[7]
Dendrobatidae	*Ameerega picta*	–	BRO -	–	[8]
Hylidae	*Bokermannohyla alvarengai*	PAS +, AB +	BRO 0	SUBB -	[9]
	Hyla cinerea	PAS +	–	–	[10]
	H. arborea	–	–	Nile blue -, Potassium dichromate and osmium tetroxide -	[11]
	various *Hyla*	PAS +, AB-1 -, AB-2.5 +	–	OIO -, SUBB - (secretion +)	[12]
	Litoria caerulea	PAS +	–	–	[13, 14]
Leiuperidae	various *Pleurodema*, *Somuncuria somuncurensis*	PAS-HE +, AB-2.5 +	NIN -	–	[15]
Leptodactylidae	*Leptodactylus lineatus*	–	BRO -	–	[8]
Pipidae	*Xenopus laevis*	PAS-AB-2.5 +, PAS +, AB-2.5 +	NIN -, Performic acid-AB +	SUBB -, Copper Phtalocyanin -	[16]
Phyllomedusidae	*Phyllomedusa distincta*	PAS-AB-2.5 +	BRO -	–	[17]
	P. hypochondrialis	PAS +, AB-1 -, AB-2.5 +	–	OIO -, SUBB - (secretion +)	[12]
Ranidae	various *Phrynobatrachus*	PAS +, AB-0.2 +	–	–	[18]
	Rana pipiens	PAS +, AB-0.4 +, AB-2.8 +	BRO -	–	[19]
	R. utricularia	PAS +, AB-1 -, AB-2.5 +	–	OIO -, SUBB - (secretion +)	[12]
	R. fuscigula	PAS +, PAS-AB +	–	–	[20]
	various *Rana*	PAS +, AB-2.5 +	NIN +	–	[21]
Rhacophoridae	*Polypedutes muculatus*	PAS +, PAS-D +, PAS (acetylated) -, PAS-AB +, AB-2.5 -/+	–	SUBB +, Br-SUBB +, SUIII -, Nile blue sulphate +	[15]
Amphiumidae	*Amphiuma tridactylum*	PAS-AB-2.5 +, AB-2.5 +	BRO 0	–	[22]
Plethodontidae	*Ensatina eschscholtzii*	PAS +, AB-2.5 -	–	–	[23]
Salamandridae	*Tylototriton verrucosus*	PAS +, AB-2.5 +	COO -	–	[24]
Siphonopidae	*Siphonops annulatus*	PAS +, AB-2.5 +	BRO -	SUB +	[25]

Staining result: - Negative, 0 Equivocal, + Positive.
Staining method: PAS Periodic acid-Schiff, AB-X Alcian blue (-pH), TOB Toluidine blue, SUBB Sudan black B, SUX Sudan I to IV, SURIII Sudan red III, OIO Oil red O, HE-EO Hematoxylin-eosin.

5.2 Anuran gland cluster reports in literature

Table SI.6 Depictions and mentions of digital mucus gland clustering in neobatrachians.

Family	Species	Reference
Centrolenidae	*Centrolenella parabambae*	[26]
Dendrobatidae	*Dendrobates tinctorius*	[26]
	Phyllobates latinasus	[26]
Hemiphractidae	*Acris gryllus*	[26]
	Gastrotheca christiani	[27]
	G. marsupiata	[28]
Hylidae	*Hyla cinerea*	This study
	Hypsiboas riojanus	[29]
	Litoria caerulea	Personal observation, [30]
	Phyllomedusa sauvagii	[27]
	P. trinitatis	[31]
	Scinax fuscovarius	[32]
Hylodidae	*Elosia bufonia*	[26]
Hyperoliidae	*Leptopelis karissimbenses*	[33]
Mantellidae	*Guibemantis timidus*	[27]
Phrynobatrachidae	*Phrynobatrachus dendrobates*	[26]
Ranidae	*Rana temporalis*	[34]
Rhacophoridae	*Philautus annandalii*	[35]
	Polypedates reinwardtii	[36]

Author details

[1]Experimental Zoology Group, Department of Animal Sciences, Wageningen University & Research, De Elst 1, 6708 WD Wageningen, The Netherlands. [2]Department of Polymer Science, The University of Akron, 170 University Ave, Ohio 44325-3909 Akron, The United States of America. [3]Biomimicry Research & Innovation Center, Departments of Biology and Polymer Science, The University of Akron, 235 Carroll St., Ohio 44325-3908 Akron, The United States of America.

References

1. Kiernan JA. Histological and Histochemical Methods: Theory and Practice. 5th ed. Banbury, UK: Scion; 2015.
2. Romeis B. Mikroskopische Technik. 16th ed. Müchen, Germany: R. Oldenbourg; 1968.
3. Mulisch M, Welsch U, editors. Romeis - Mikroskopische Technik. 18th ed. Heidelberg, Germany: Springer Spektrum; 2010.
4. Hornatowska J. Visualisation of pectins and proteins by microscopy. STFI-Packforsk; 2005.
5. Bancroft JD, Stevens A, editors. Theory and practice of histological techniques. 4th ed. New York, USA: Churchill Livingstone; 1996.
6. Lillie R, Ashburn LL. Supersaturated solutions of fat stains in dilute isopropanol for demonstration of acute fatty degeneration not shown by Herxheimer's technique. Archives of Pathology & Laboratory Medicine. 1943;36:432–440.
7. Regueira E, Dávila C, Hermida GN. Morphological Changes in Skin Glands During Development in *Rhinella Arenarum* (Anura: Bufonidae). The Anatomical Record. 2016;299:141–156.
8. Prates I, Antoniazzi MM, Sciani JM, Pimenta DC, Toledo LF, Haddad CFB, et al. Skin Glands, Poison and Mimicry in Dendrobatid and Leptodactylid Amphibians. Journal of Morphology. 2012;273:279–290.
9. Centeno FC, Antoniazzi MM, Andrade DV, Kodama RT, Sciani JM, Pimenta DC, et al. Anuran Skin and Basking Behavior: the Case of the Treefrog *Bokermannohyla alvarengai* (Bokermann, 1956). Journal of Morphology. 2015;276:1172–1182.
10. Ernst VV. The digital pads of the tree frog, *Hyla cinerea*. II. The mucous glands. Tissue and Cell. 1973;5(1):97–104.
11. Goniakowska-Witalińska L, Kubiczek U. The structure of the skin of the tree frog (*Hyla arborea arborea* L.). Annals of Anatomy. 1998;180:237–246.
12. Barbeau TR, Lillywhite HB. Body wiping behaviors associated with cutaneous lipids in hylid tree frogs of Florida. Journal of Experimental Biology. 2005;208:2147–2156.
13. Warburg MR, Rosenberg M, Roberts JR, Heatwole H. Cutaneous glands in the Australian hylid *Litoria caerulea* (Amphibia, Hylidae). Anatomy and Embryology. 2000;201(5):341–348.
14. Barnes WJP, Perez Goodwyn PJ, Nokhbatolfoghahai M, Gorb SN. Elastic modulus of tree frog adhesive toe pads. Journal of Comparative Physiology A. 2011;197(10):969–978.
15. Ferraro DP, Topa PE, Hermida GN. Lumbar glands in the frog genera *Pleurodema* and *Somuncuria* (Anura: Leiuperidae): histological and histochemical perspectives. Acta Zoologica. 2011;00:1–14.
16. Thomas EO, Tsang L, Licht P. Comparative Histochemistry of the Sexually Dimorphic Skin Glands of Anuran Amphibians. Copeia. 1993;1993(1):133–143.
17. Antoniazzi MM, Neves PR, Mailho-Fontana PL, Rodrigues MT, Jared C. Morphology of the parotoid macroglands in *Phyllomedusa* leaf frogs. Journal of Zoology. 2013;291:42–50.
18. Le Quang Trong Y. Étude de la peau et des glandes cutanées de quelques Amphibiens du genre *Phrynobatrachus*. Bulletin de l'Institut français d'Afrique noire, Série A. 1971;33:987–1025.
19. Dapson RW. Histochemistry of Mucus in the Skin of the Frog, *Rana pipiens*. The Anatomical Record. 1969;166(4):615–625.
20. Els WJ, Henneberg R. Histological features and histochemistry of the mucous glands in ventral skin of the frog (*Rana fuscigula*). Histology and Histopathy. 1990;5:343–348.
21. Brizzi R, Delfino G, Pellegrini R. Specialized Mucous Glands and Their Possible Adaptive Role in the Males of Some Species of *Rana* (Amphibia, Anura). Journal of Morphology. 2002;254:328–341.
22. Pereira KE, Crother BI, Sever DM, Fontenot CL Jr, Pojman JA Sr, Wilburn DB, et al. Skin glands of an aquatic salamander vary in size and distribution and release antimicrobial secretions effective against chytrid fungal pathogens. Journal of Experimental Biology. 2018;221.
23. Fontana MF, Ask KA, MacDonald RJ, Carnes AM, Staub NL. Loss of traditional mucous glands and presence of a novel mucus-producing granular gland in the plethodontid salamander *Ensatina eschscholtzii*. Biological Journal of the Linnean Society. 2006;87:469–477.
24. Wanninger M, Schwaha T, Heiss E. Form and Function of the skin glands in the Himalayan newt *Tylototriton verrucosus*. Zoological Letters. 2018;4:2–10.
25. Jared C, Mailho-Fontana PL, Marques-Porto R, Sciani JM, Carvalho Pimenta D, Brodie Jr ED, et al. Skin gland concentrations adapted to different evolutionary pressures in the head and posterior regions of the caecilian *Siphonops annulatus*. Scientific Reports. 2018;8:1–7.
26. Noble GK, Jaeckle ME. The digital pads of the tree frogs. A study of the phylogenesis of an adaptive structure. Journal of Morphology and Physiology. 1928;45(1):259–292.
27. Manzano AS, Fabrezi M, Vences M. Intercalary elements, treefrogs, and the early differentiation of a complex system in the Neobatrachia. The Anatomical Record. 2007;290(12):1551–1567.
28. Hertwig I, Sinsch U. Comparative Toe Pad Morphology in Marsupial Frogs (Genus *Gastrotheca*): Arboreal versus Ground-Dwelling Species. Copeia. 1995;1995(1):38–47.
29. Manzano AS, Fontanarrosa G, Prieto Y, Abdala V. La prensilidad en anfibios y reptiles: perspectivas evolutivas basadas en la anatomía y la función. In: Morfología de Vertebrados Conceptos, métodos y grupos de investigación en Argentina; 2017. p. 59–82.
30. Nakano M, Saino T. Light and electron microscopic analyses of the high deformability of adhesive toe pads in White's tree frog, *Litoria caerulea*. Journal of Morphology. 2016;277:1509–1516.

31. Ba-Omar TA, Downie JR, Barnes WJP. Development of adhesive toe-pads in the tree-frog (*Phyllomedusa trinitatis*). Journal of Zoology. 2000;250:267–282.
32. Fabrezi M, Goldberg J, Chuliver Pereyra M. Morphological Variation in Anuran Limbs: Constraints and Novelties. Journal of Experimental Zoology Part B: Molecular and Developmental Biology. 2017;328(6):546–574.
33. Drewes RC. A Phylogenetic Analysis of the Hyperoliidae (Anura): Treefrogs of Africa, Madagascar, and the Seychelles Islands. Occasional papers of the California Academy of Sciences. 1984;139:1–70.
34. Gaupp E. A. Ecker's und R. Wiedersheim's Anatomie des Frosches auf Grund eigener Untersuchungen durchaus neu bearbeitet. 2nd ed. Braunschweig, Germany: Vieweg und Sohn; 1904.
35. Chakraborti S, Das D, De SK, Nag TC. Structural organization of the toe pads in the amphibian *Philautus annandalii* (Boulenger, 1906). Acta Zoologica. 2014;95:63–72.
36. Siedlecki M. Die Haftballen des javanischen Flugfrosches. Bulletin of the Academy of Sciences, Krakow (B). 1910;p. 593–606.
